# NK cells with decreased expression of multiple activating receptors is a dominant phenotype in pediatric patients with acute lymphoblastic leukemia

**DOI:** 10.3389/fonc.2022.1023510

**Published:** 2022-11-07

**Authors:** Lucero Valenzuela-Vázquez, Juan Carlos Nuñez-Enriquez, Jacqueline Sánchez-Herrera, Aurora Medina-Sanson, María Luisa Pérez-Saldivar, Elva Jiménez-Hernández, Jorge Alfonso Martiín-Trejo, María de Los Ángeles Del Campo-Martínez, Janet Flores-Lujano, Raquel Amador-Sánchez, Félix Gustavo Mora-Ríos, José Gabriel Peñaloza-González, David Aldebarán Duarte-Rodríguez, José Refugio Torres-Nava, Rosa Martha Espinosa-Elizondo, Beatriz Cortés-Herrera, Luz Victoria Flores-Villegas, Laura Elizabeth Merino-Pasaye, Carolina Almeida-Hernández, Rosario Ramírez-Colorado, Karina Anastacia Solís-Labastida, Francisco Medrano-López, Jessica Arleet Pérez-Gómez, Martha Margarita Velázquez-Aviña, Annel Martínez-Ríos, Antonio Aguilar-De los Santos, Jessica Denisse Santillán-Juárez, Alma Gurrola-Silva, Alejandra Jimena García-Velázquez, Minerva Mata-Rocha, Gabriela Alicia Hernández-Echáurregui, Omar Alejandro Sepúlveda-Robles, Haydeé Rosas-Vargas, Ismael Mancilla-Herrera, Silvia Jimenez-Morales, Alfredo Hidalgo-Miranda, Ivan Martinez-Duncker, Jeremy D. Waight, Kenneth W. Hance, Kevin P. Madauss, Juan Manuel Mejía-Aranguré, Mario Ernesto Cruz-Munoz

**Affiliations:** ^1^ Facultad de Medicina, Universidad Autónoma del Estado de Morelos, Cuernavaca, Morelos, Mexico; ^2^ Instituto de Investigación en Ciencias Básicas y Aplicadas, Universidad Autónoma del Estado de Morelos, Cuernavaca, Morelos, Mexico; ^3^ Unidad de Investigación Médica en Epidemiología Clínica, Unidad Médica de Alta Especialidad (UMAE) Hospital de Pediatría, Centro Médico Nacional (CMN) “Siglo XXI”, Instituto Mexicano del Seguro Social (IMSS), Mexico City, Mexico; ^4^ Servicio de Oncología Pediátrica, Hospital Infantil de México, “Dr. Federico Gómez Sántos”, Secretaria de Salud, Ciudad de México, Mexico; ^5^ Servicio de Hematología Pediátrica, Hospital General “Gaudencio González Garza”, Centro Médico Nacional (CMN) “La Raza”, Instituto Mexicano del Seguro Social (IMSS), Mexico City, Mexico; ^6^ Servicio de Hematología Pediátrica, Unidad Médica de Alta Especialidad (UMAE) Hospital de Pediatría, Centro Médico Nacional (CMN) “Siglo XXI”, Instituto Mexicano del Seguro Social (IMSS), Mexico City, Mexico; ^7^ Hospital General Regional No. 1 “Carlos McGregor Sánchez Navarro”, Instituto Mexicano del Seguro Social (IMSS), Mexico City, Mexico; ^8^ Departamento de Hematología, Hospital General Regional Ignacio Zaragoza del Instituto de Seguridad y Servicios Sociales de los Trabajadores del Estado (ISSSTE), Mexico City, Mexico; ^9^ Servicio de Onco-Pediatria, Hospital Juárez de México, Secretaria de Salud (SS), Mexico City, Mexico; ^10^ Servicio de Oncología, Hospital Pediátrico de Moctezuma, Secretaría de Salud de la Ciudad de México (CDMX), Mexico City, Mexico; ^11^ Servicio de Hematología Pediátrica, Hospital General de México, Secretaria de Salud (SS), Mexico City, Mexico; ^12^ Servicio de Hematología Pediátrica, Centro Médico Nacional (CMN) “20 de Noviembre”, Instituto de Seguridad Social al Servicio de los Trabajadores del Estado (ISSSTE), Mexico City, Mexico; ^13^ Hospital General de Ecatepec “Las Américas”, Instituto de Salud del Estado de México (ISEM), Mexico City, Mexico; ^14^ Hospital Pediátrico La Villa, Secretaría de Salud de la Ciudad de México (SSCDMX), Mexico City, Mexico; ^15^ Hospital General Regional (HGR) No. 72 “Dr. Vicente Santos Guajardo”, Instituto Mexicano del Seguro Social (IMSS), Mexico City, Mexico; ^16^ Hospital General de Zona 98, Instituto Mexicano del Seguro Social (IMSS), Mexico City, Mexico; ^17^ Servicio de Hemato-oncología Pediátrica, Hospital Regional No. 1° de Octubre, Instituto de Seguridad y Servicios Sociales de los Trabajadores del Estado (ISSSTE), Mexico City, Mexico; ^18^ Hospital Regional Tipo B de Alta Especialidad Bicentenario de la Independencia, Instituto de Seguridad Social al Servicio de los Trabajadores del Estado, Mexico City, Mexico; ^19^ Unidad de Investigación Médica en Genética Humana, Unidad Médica de Alta Especialidad (UMAE) Hospital de Pediatría, Centro Médico Nacional (CMN) “Siglo XXI”, Instituto Mexicano del Seguro Social (IMSS), Mexico City, Mexico; ^20^ Departamento de Infectología e Inmunología, Instituto Nacional de Perinatología, Mexico City, Mexico; ^21^ Laboratorio de Genómica del Cáncer, Instituto Nacional de Medicina Genómica, Mexico City, Mexico; ^22^ Centro de Investigación en Dinámica Celular, Universidad Autónoma del Estado de Morelos, Cuernavaca, Morelos, Mexico; ^23^ Oncology R&D, GlaxoSmithKline, Collegeville, PA, United States; ^24^ Facultad de Medicina, Universidad Nacional Autónoma de México, Mexico City, Mexico

**Keywords:** NK cells, acute lymphoblastic leukemia, immunooncology, cancer, immune system

## Abstract

NK cells have unique attributes to react towards cells undergoing malignant transformation or viral infection. This reactivity is regulated by activating or inhibitory germline encoded receptors. An impaired NK cell function may result from an aberrant expression of such receptors, a condition often seen in patients with hematological cancers. Acute lymphoblastic leukemia (ALL) is the most common pediatric cancer worldwide and NK cells have emerged as crucial targets for developing immunotherapies. However, there are important gaps concerning the phenotype and behavior of NK cells during emergence of ALL. In this study we analyze the phenotype and function of NK cells from peripheral blood in pediatric patients with ALL at diagnosis. Our results showed that NK cells exhibited an altered phenotype highlighted by a significant reduction in the overall expression and percent representation of activating receptors compared to age-matched controls. No significant differences were found for the expression of inhibitory receptors. Moreover, NK cells with a concurrent reduced expression in various activating receptors, was the dominant phenotype among patients. An alteration in the relative frequencies of NK cells expressing NKG2A and CD57 within the mature NK cell pool was also observed. In addition, NK cells from patients displayed a significant reduction in the ability to sustain antibody-dependent cellular cytotoxicity (ADCC). Finally, an aberrant expression of activating receptors is associated with the phenomenon of leukemia during childhood.

## Introduction

Acute lymphoblastic leukemia (ALL) is the most common pediatric cancer worldwide and follows a lethal course if an effective treatment is not provided (1). Whereas the causal mechanisms of ALL are not well known, the whole series of intrinsic and extrinsic factors that contribute or influence the course of the disease, are neither fully explored. A link between immune system and cancer is based on the ability of immune cells to recognize and eliminate cancer cells (2, 3). A failure in the mechanisms by which immune cells recognize and eliminate malignant cells may favor the emergence of cancer. Whereas this condition is well described and documented in solid tumors, much less is known in liquid cancers such as ALL (4). Relative to other immune populations, NK cells play a dominant role in recognizing and eliminating leukemia cells (5). In agreement with this idea, improved cure rates of high-risk pediatric ALL patients have been observed following hematopoietic stem cell transplantation (HSCT) from donors with alloreactive NK cells (6–8). Furthermore, NK cells arising from the grafted hematopoietic precursors, were found to be responsible for a favorable clinical outcome (9). Therefore, NK cells are key mediators of immunosurveillance and they have emerged as an important immune population targeted for cancer immunotherapy (10–12). Despite the potential for NK cells in the treatment of acute leukemia (13), several studies have documented that ALL blast are not effectively eliminated by NK cells (14–17). Whereas the precise mechanisms underlying this phenomenon remain poorly understood, it is proved that the numbers and function of NK cells are compromised (17–20).

NK cells recognize and destroy malignant cells by surveying for a lack of cell surface major histocompatibility complex (MHC) class I molecules (‘missing-self’), as well as *de novo* appearance of molecules on target cells (‘induced-self’) (21). NK cell recognition and reactivity are mediated by various germline encoded receptors that either stimulate or restrain NK cell effector functions (21, 22). Among receptors with activating functions for NK cells are those belonging to the Natural Cytotoxicity Receptors (NCR) family, Signaling Lymphocyte Activation Molecule (SLAM) family, Natural Killer Group (NKG) (23), and Immunoglobulin (Ig)-like receptors such as DNAM-1 (CD226) (24, 25). By contrast, receptors grouped in the NK cell Ig-like receptors (KIR) family, are responsible for recognizing “self”, and to provide inhibitory signals to prevent unsolicited NK cell activation (26). In addition to inhibitory KIRs, others receptors have been shown to dampen NK cell function, including TIGIT, CD96, TIM-3, and PD-1 (27, 28). The expression density of activating and inhibitory receptors may also influence NK cell reactivity. Indeed, defective NK cell effector functions may result from aberrant expression of receptors, a phenomenon often seen in patients with various types of cancers and other pathologies (29–33). Recent studies, have also suggested a role for inhibitory checkpoints in mediate immune suppression (34), however their contribution in an abnormal NK cell function during ALL remains elusive.

In this study, we demonstrate for the first time that in a cohort of 72 pediatric ALL patients, NK cells from peripheral blood exhibited a reduced overall expression of various activating receptors at diagnosis relative to age-matched healthy controls. By contrast, we did not find any significant difference between patients and controls regarding the expression of inhibitory receptors. Moreover, among ALL patients, the frequencies of NK cells with a decreased expression in more than one single-type of activating receptors was dominant over NK cells with a decreased expression in just a single-type of activating receptors. In addition, a significant change in the relative proportions of NK cells expressing NKG2A and CD57 in the mature NK-cell pool was observed in ALL patients compared to healthy controls. Moreover, NK cells from patients displayed a significant reduction in reverse antibody dependent cell cytotoxicity (rADCC) compared to healthy controls. Finally, a logistical regression analysis demonstrated that an abnormal expression of activating receptors in NK cells is associated with an increased risk of developing childhood ALL, suggesting a protective role of activating receptors against acute leukemia.

## Materials and methods

### Patients and study design

This study was conducted under two different designs. The first, is a case-control study that included 72 patients with newly diagnosed ALL, and 21 age-matched healthy controls (**Table 1**). A second cohort study, that included the same 72 ALL patients, was designed for identifying possible associations between the NK cell phenotype and the risk of acute lymphoblastic leukemia. Peripheral blood samples were collected from incident cases of ALL treated in public hospitals in Mexico City under 18 years of age. Diagnosis of ALL was based on the morphologic and immunophenotypic features of leukemic cells. Peripheral blood samples (2–3 ml) from patients were obtained at diagnosis and before treatment initiation. Healthy controls were matched with cases according to gender frequencies and age. The exclusion criteria for controls were neoplasms, hematological diseases, and congenital malformations. Allergies and infections were an exclusion criteria if they were the reason for the hospital admission at the time of recruitment. An approval by National Scientific Research and Ethics Committee was obtained with the number R-2016-785-042.

**Table 1:** Clinical characteristic of the validation cohort.

Study variables	Controls	ALL cases
	n=21	n=72
	n (%)	n (%)
**Child’s sex**		
Female	10 (47.6)	32 (44.4)
Male	11 (52.4)	40 (55.6)
**Age group (years)**		
< 1	1 (4.7)	1 (1.4)
1-9	11 (52.4)	42 (58.3)
≥ 10	9 (42.9)	29 (40.3)
**Infections in the first year of life**		
No	8 (38.1)	26 (36.1)
Yes	13 (61.9)	46 (63.9)
**Allergies**		
No	13 (61.9)	60 (83.3)
Yes	8 (38.1)	12 (16.7)
**Leukocyte Count**		
< 50,000	-----	58 (80.6)
≥ 50,000	-----	14 (19.4)
**Rearrangement**		
Not Detected	-----	67 (93.1)
Detected	-----	5 (6.9)
**Immunophenotype**		
Pre-B	-----	70 (97.2)
T cell	-----	2 (2.8)
**NCI Risk**		
High Risk		37 (51.4)
Standard Risk		35 (48.6)
**Relapse**		
No	-----	65 (90.3)
Yes	-----	7 (9.7)
**Death**		
No	-----	64 (88.9)
Yes	-----	8 (11.1)

ALL, acute lymphoblastic leukemia.

The median follow-up time of the patient cohort was 10 months (4-19 months) after confirmation of the diagnosis.

### Clinical data collection and risk classification

Risk classification at diagnosis was based on the National Cancer Institute risk criteria. All patients were treated according to the chemotherapy protocol used in each hospital where patients received medical care. For the case-control study, variables as infection during the first year of age and allergies during the infancy were registered as confounding variables. The medical history of cases and controls regarding common infections in the first year of life and allergies was explored as it has been recommended and used in previous studies (35, 36). In relation to common infections, we interrogated for any infections (including routine childhood viral infections, non-severe infections such as acute otitis, serious infections causing hospitalization, among other common infections). Concerning the allergies, the cases’ parents were required to indicate whether their children had any allergy for at least 1 year before the diagnosis of ALL, whereas the control children’s parents were asked about the occurrence of any allergy at least 1 year before the interview. The parents were questioned about the specific type of allergy (asthma, rhinitis, skin allergy, and other allergies such as seasonal, food, drug, etc.).

### Antibodies and reagents

The antibodies used in this study are listed in **Supplementary Table 1**.

### NK cell phenotyping

Peripheral blood was collected in EDTA-containing vacutainers. Then, 50 μl of blood was aliquoted in three different tubes and incubated with a different combination of primary antibodies (Ab) for 30 min at 4°C (**Supplementary Table 1**). Then, cells were washed, red blood cells lysed with 1X lysis buffer (BD Pharm Lyse™ Cat: 555899) and, fixed with 2% paraformaldehyde for subsequent acquisition. Autofluorescence was used as a negative control, previously validated with isotype controls using the same combination of fluorochromes. The samples were acquired in a FACSCanto II cytometer (BD bioscience). NK cells were defined as CD3-CD20-CD14-CD56+ cells. Final analysis of the expression for each NK cell marker was performed using FlowJo 7.6.5 software (Tree Star, Ashland, OR) and Infinicyt 2.0.4.

### NK cell degranulation assays

NK cell degranulation assays were performed as elsewhere described (37, 38). Briefly, PBMCs (1x10^6^/ml) were incubated with P815 cells (2x10^6^/ml) in a total volume of 200 μl in a 96 well plate. P815 cells were supplemented with 5mg/ml of anti-CD16 mAb (Clone 3G8, eBiosciences). After 3 hours of incubation at 37°C, cells were recovered and stained using following antibodies: anti-CD3 FITC, anti-CD56 APC, and anti-CD107 PE (Biolegend, clone H4A3). GolgiStop was not included in these assays. Cells were acquired on FACSCanto II (BD bioscience) and analyses with the use of FlowJo 7.6.5 software (Tree Star, Ashland, OR). Gates were set to exclude CD3+ lymphocytes. Thereafter, the percentage of cells positive for CD107a was obtained after gating in CD3-CD56+ lymphocytes. The basal percentages for CD107a were obtained from PBMCs incubated with P815 cells with no agonist mAb. Degranulation was represented as the fold increase for CD107a, which is the difference between the percentage of NK cells expressing CD107a at surface after stimulation with P815 cells supplemented with agonist mAb, and the percentage of NK cells expressing CD107a at NK cell surface after incubation with P815 cells with no agonist mAb. NK cell degranulation assays were performed in 32 acute leukemia patients and 10 age-matched healthy controls.

### Statistical analysis

The analysis was conducted using SPSS software (version 21; IBM Statistical Package for the Social Sciences, Chicago, IL). A descriptive analysis was performed calculating frequencies and percentages for the categorical variables. Likewise, the distribution of continuous variables was evaluated using Shapiro-Wilk. The percentages on NK cells expressing a given surface marker and its relative expression displayed a non-parametric distribution. The difference in the expression of each receptor between cases and controls was evaluated using the Mann Whitney U one-tailed test. A value of p <0.05 was considered statistically significant. Furthermore, the expression of the immunoreceptors was categorized into two groups according to the 50th percentile of the expression observed in the control group. Subsequently, the 95% confidence interval (CI), the unadjusted odds ratios (OR) and adjusted odds ratios (aOR), were calculated using unconditional logistic regression analysis. Adjusting variables were the child´s age, gender, infections during the first year of life, and a history of allergies. In this study, infections and allergies were considered as confounding variables since an early life exposure to infectious agents and allergies are two well-known proxies of the immune stimulation process that have been associated with acute lymphoblastic leukemia during childhood (36, 39). Additionally, associations between immunoreceptors and clinical characteristics (age >10 years, male sex, history of allergies, >50,000xmm3 leukocyte count and T cell immunophenotype) and outcomes (relapse, death) were explored in the group of cases with ALL. For Venn diagrams we use the Eulerr package for R version 6.1.0.

## Results

### Demographic and clinical features of pediatric ALL patients

In this study, we characterized peripheral blood NK cells from 72 ALL patients, collected at diagnosis, along 21 age-matched healthy controls. In the present study no significant difference was observed between cases and controls regarding child’s sex and age (**Table 1**). B cell (B)-ALL was the predominant disease subtype, accounting for 97.2% of all patient samples analyzed, with 6.9% of total patients harboring a genetic rearrangement. 14 patients (19.4%) presented with leukocyte counts >50,000 x mm^3^, 7 patients (9.7%) relapsed, and 8 patients (11.1%) died (**Table 1**). Infections during the first year of life was found to be similar between control and ALL groups (61.9% and 63.9%, respectively), while allergies were more frequent in the group control (38.1% and 16.7%, respectively) (**Table 1**).

### Decreased expression of activating receptors in NK cells from patients with acute leukemia

**Figure 1:**
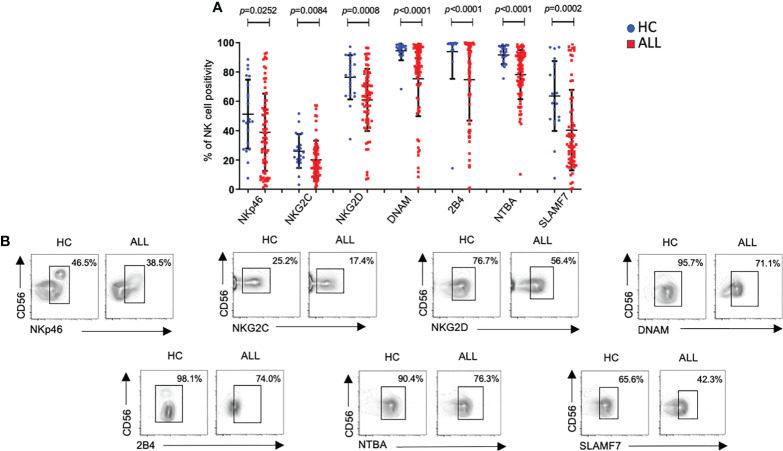
Percentages of NK cells expressing distinct activating receptors from ALL patients and healthy controls. **(A)** The frequencies of CD3^-^CD20^-^CD14^-^CD56^+^ cells expressing distinct activating receptors were analyzed by flow cytometry in 72 ALL patients and 21 healthy controls. Vertical lines indicate standard deviation. The horizonal lines represents the mean value. P values reports significance according to U-Mann Whitney Test one-tail. HC: healthy controls; ALL: Acute lymphoblastic leukemia patients. **(B)** Representative FACS plots depict the percentage of CD3^-^CD20^-^CD14^-^CD56^+^ cells expressing distinct activating receptors from healthy controls (*n* = 21) and ALL patients (*n* = 72). HC: healthy controls; ALL: Acute lymphoblastic leukemia patients.

Before analyzing the phenotype on peripheral blood NK cells, we compared the relative frequencies of NK cells between patients and controls. The relative frequencies of the two major NK cell subsets (CD56 dim and CD56 bright) were significantly reduced in pediatric ALL patients compared with age-matched healthy controls (P<0.0001) (**Supplementary Figures 1A, B**). Then, we analyzed the expression frequency of major receptors associated with NK cell activation. As reported before (17, 24), we observed a significant decrease (*P*=0.0252) in the percentage of NKp46^+^ NK cells in ALL patients compared with controls (**Figure 1A**). A significant decrease in the percentages of NKG2C^+^NK cells (*P*=0.0084) and NKG2D^+^ NK cells (*P*=0.0008) was also found in ALL patients when compared with healthy controls (**Figure 1A**). DNAM showed also a significant decrease (*P*=<0.0001) in the percentage of DNAM-1^+^ NK cells in ALL patients when compared with healthy controls (**Figure 1A**). Finally, the relative frequencies of NK cells expressing members of the SLAM including 2B4 (SLAMF4), NTBA (SLAMF6), or SLAMF7, were also significantly decreased (2B4^+^ *P*<0.0001; NTBA^+^ *P*<0.0001; SLAMF7^+^ *P*=0.0002) in ALL patients relative to age-matched controls. Representative flow cytometer plots from controls and patients are shown in **Figure 1B**.

**Figure 2:**
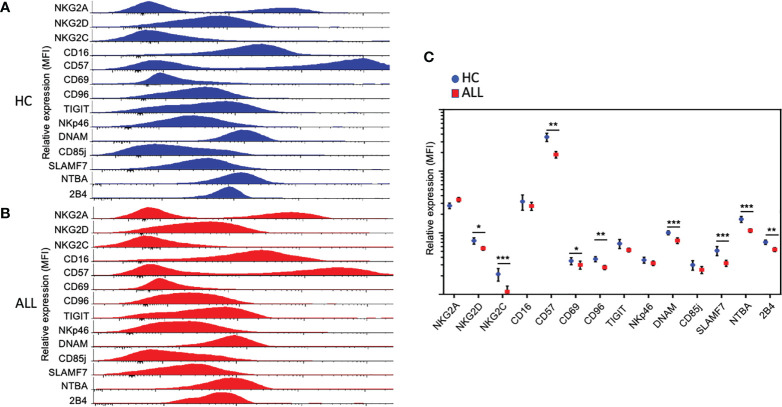
Relative expression of activating and inhibitory receptors on NK cells. Relative expression, represented as the mean fluorescence intensity (MFI), of activating and inhibitory receptors in NK cells from total healthy controls (n = 21) **(A)** and ALL patients (n = 62) **(B)**. Histograms cover all the values obtained for each control and patient as indicated. **(C)** Summary of data for frequencies of NK cell expressing distinct activating and inhibitory receptors from21 healthy controls and 62 ALL patients. The vertical lines indicate standard deviation. The horizonal lines represent the mean value. Results were considered significant at * *p*< 0.05, ** p < 0.01 and *** p < 0.001.

We analyzed the percentage of NK cells expressing markers associated with alternative lymphocyte and NK cell functions, including differentiation and cellular retention. For these markers, we did not observe a significant difference in the percentage of CD16+ NK cells in ALL patients when compared with controls (**Supplementary Figures 2A, B**). Like CD16, we observed no significant difference in the percentage of NK cells expressing CD69 (**Supplementary Figures 2A, B**). Finally, we also assessed the expression percentage of CD57, a marker associated with replicative senescence or terminal differentiation (40–42). The percentage of CD57^+^ NK cells was significantly reduced (*P*=0.0175) in ALL patients relative to healthy controls. Consistent with a reduced percentage positivity, we found lower overall expression density of NKG2C, DNAM-1, SLAMF7, NBTA, 2B4, and CD57 (**Figures 2A–C**). Interestingly, despite the lack of overt differences in percent positivity, we found lower overall expression density of CD69 on NK cells. Altogether, the data suggest that NK cells from pediatric ALL patients display an aberrant NK cell phenotype characterized by a significant reduction in the percent positivity and overall expression density of various activating receptors.

### NK cells from pediatric ALL patients exhibit normal inhibitory receptor expression

**Figure 3:**
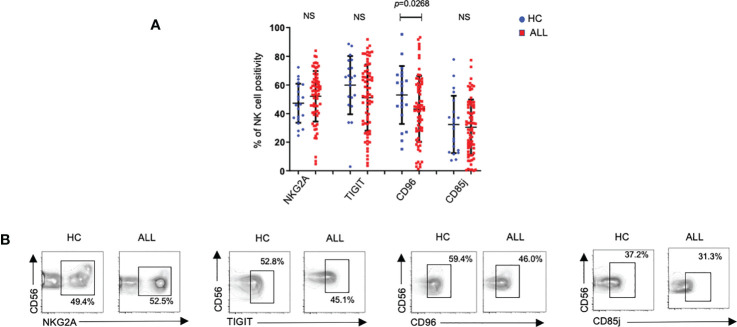
Percentages of NK cells expressing distinct inhibitory receptors on NK cells form healthy controls and ALL patients. **(A)** The frequencies of CD3^-^CD20^-^C14^-^CD56^+^ cells expressing distinct inhibitory receptors were analyzed by flow cytometry in 72 ALL patients and 21 healthy controls. Vertical lines indicate standard deviation. The horizonal lines represents the mean value. **(B)** Representative FACS plots depict the percentage of CD3^-^CD20^-^CD14^-^CD56^+^ expressing distinct inhibitory receptors on NK cells from healthy controls (*n*= 21) and ALL patients (*n*= 72). HC: healthy controls; ALL: Acute lymphoblastic leukemia patients. NS, Not Significant.

Alongside inhibitory receptors belonging to the KIR family, several recently-described receptors have been ascribed broad immunoregulatory roles (27). One of such receptors, TIGIT, has been shown to inhibit the effector function of both T and NK cells (43–45). With this in mind, we evaluated the expression frequency of TIGIT on peripheral blood NK cells from pediatric ALL patients. While we failed to observe significant differences in TIGIT^+^ NK cells between ALL and healthy donor samples, we found a significant decrease in CD96, another member of the DNAM-1-TIGIT axis (46) (**Figure 3**). NKG2A, is an inhibitory receptor which expression is upregulated in patients with acute leukemia (17). No significant differences were observed for the percentage of NKG2A^+^ NK cells between patients and controls (**Figure 3**). Finally, we also evaluated the expression frequency of CD85j (ILT2), an immunoglobulin (Ig)-like receptor, which displays an inhibitory role in NK cells upon ligation with HLA molecules (47). As with TIGIT and NKG2A, we did not find any significant differences in the percentage of NK cells expressing CD85j in ALL patients when compared with controls. When the overall expression of NKG2A, TIGIT, and, CD85j was analyzed, we did not find any significant difference between healthy controls and ALL patients (**Figures 2A–C**). As an exception to these findings, a significant decrease in the expression density of CD96 was observed in ALL patients when compared to healthy controls. These data suggest that in contrast to broad modulation of activating receptors, NK cells from pediatric ALL patients do not display an irregular inhibitory receptor expression profile.

### A convergent decreased expression of activating receptors dominates the NK cell phenotype among ALL patients

**Figure 4:**
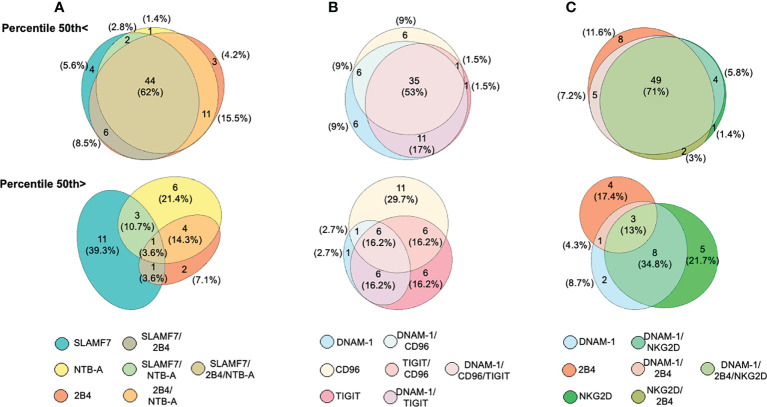
Analysis of concurrent expression of receptors in NK cells among ALL patients. Venn diagrams showing the number of functions, as defined by Boolean gating, simultaneously exhibited by NK cells in patients below the 50th percentile (top panels) or above the 50th percentile (bottom panel) for SLAM, 2B4 and, NTB-A **(A)**, DNAM-1, TIGIT and, CD96 **(B)**, DNAM-1, 2B4 and, NKG2D **(C)**. The seven possible combinations are represented with a different color and the number of patients having one of these possible combinations are indicated as percentages.

Since a significant reduction in the expression frequency of various NK cell activating receptors was observed in ALL patients when compared with healthy controls, we analyzed the concurrent expression patterns for such activating receptors among NK cells from ALL patients. Patients were distributed in two categories based on the 50th percentile. Seven different possible phenotype combinations were analyzed and their profiles were analyzed by the Eulerr package for R version 6.1.0. (**Figure 4**). We found that in 62% (n=44) of patients with ALL, NK cells had a convergent decreased expression frequency for SLAMF7, 2B4 and, NTB-A (**Figure 4A**, top panel). In contrast, only one patient (3.6%) display a normal concurrent expression for such activating receptors (**Figure 4A** bottom panel). A concurrent decreased expression frequency for DNAM-1, TIGIT and, CD96 in NK cells was observed in 53% of ALL patients (**Figure 4B**, top panel), whereas 16.2% of ALL patients showed NK cells with a normal concurrent for DNAM-1, TIGIT and, CD96 (**Figure 4B**, bottom panel). Finally, when we analyzed the expression profile for DNAM-1, 2B4 and, NKG2D, we found that in 71% of ALL patients, NK cells displayed a concurrent decrease expression frequency for DNAM-1, 2B4 and, NKG2D (**Figure 4C**, top panel), whereas only 13% of ALL patients presented a normal concurrent expression for these activating receptors (**Figure 4C**, bottom panel). Therefore, these data suggest that among ALL patients, the percentages of NK cells with a decreased concomitant expression for various activating receptors either belonging to a single family or multiple families was dominant over NK cells displaying a significant reduction is just a single type of receptor.

### NK cells from patients with pediatric ALL display an abnormal differentiation phenotype in the mature NK-cell pool

**Figure 5:**
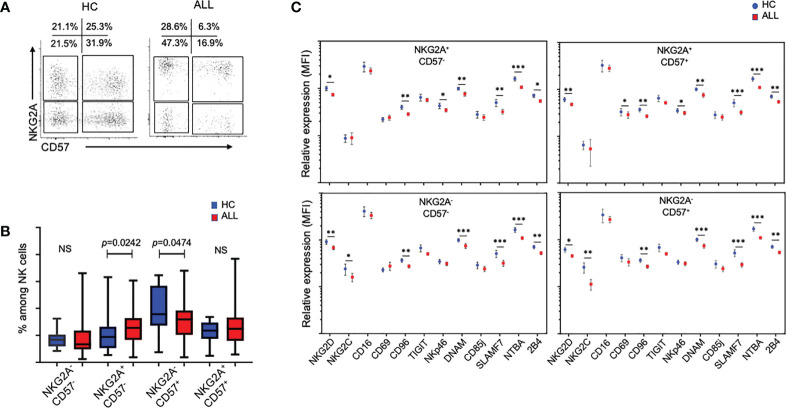
Analysis of in the mature NK-cell pool from ALL patients and healthy controls. **(A)** Representative FACS plots of CD3^-^CD20^-^C14^-^CD56^+^ cells at different stages of maturation according to the expression of NKG2A and CD57 from healthy controls and ALL patients. **(B)** Summary of data for frequencies of CD3^-^CD20^-^C14^-^CD56^+^ cells according to the expression NKG2A and CD57. Vertical lines indicate standard deviation. The horizonal lines represents the mean value. P values reports significance according to U-Mann Whitney Test one-tail. HC, healthy controls; ALL, Acute lymphoblastic leukemia patients. **(C)** Summary of data for relative expression, represented as the mean fluorescence intensity (MFI), of CD3^-^CD20^-^C14^-^CD56^+^ cells at different stages of maturation according to expression of NKG2A and CD57. The vertical lines indicate standard deviation. HC, healthy controls; ALL, Acute lymphoblastic leukemia patients. The horizonal lines represents the mean value. Results were considered significant at *p < 0.05, **p < 0.01 and ***p < 0.001. NS, Not Significant.

Loss of NKG2A expression, accompanied by acquisition of CD57 and KIRs, has been shown to be useful in determining the differentiation status of NK cells (19–22). Using these markers, we investigated the relative impact of ALL on NKG2A/CD57-delineated NK cell subsets. Interestingly, relative to healthy donor controls, the percentage of NKG2A^+^CD57^-^ (immature-like) NK cells were significantly increased (*P*=0.0242), whereas NKG2A^-^CD57^+^ (mature) NK cells were significantly decreased (*P*=0.0474), suggesting that patients with ALL exhibit defects in NK cell terminal differentiation (**Figures 5A, B**). Based on these findings, we decided to analyze the relative expression of activating and inhibitory receptors in the four major NK cell subsets distinguished by the differential expression of NKG2A and CD57. As shown in **Figure 5C**, the downregulation of NKG2D, CD96, DNAM-1, SLAMF7, NTBA, and 2B4 was significant consistent through the four major subsets of the peripheral NK cell pool. These data suggest that irrespective of the heterogeneity of subsets observed in the NK cell pool, activating receptors are broadly modulated in pediatric ALL patients.

### NK cells from ALL patients exhibit an abnormal cytotoxicity

**Figure 6:**
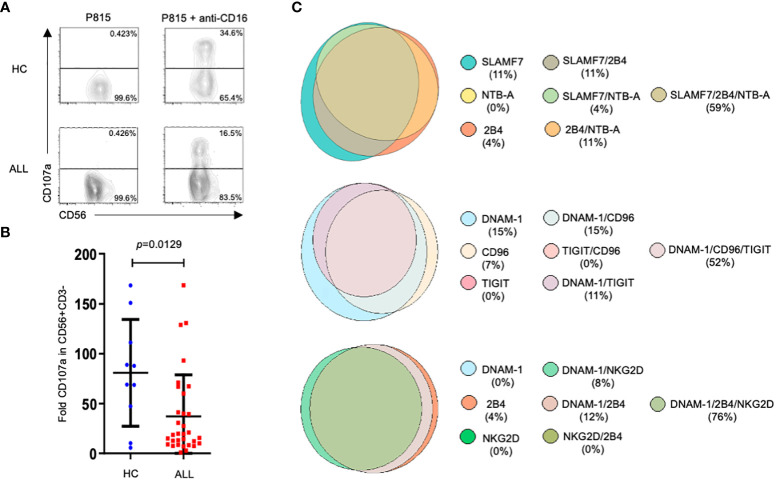
Degranulation assays in NK cells from ALL patients and healthy controls. Representative FACS plots depict NK cell response (CD107a degranulation) against P815 supplemented or not with anti-CD16 (clone 4G6). **(A)** PBMCs (1x106/ml) were incubated with P815 cells (2x106/ml) for 3 hours in a total volume of 200 ul in a 96 well plate at 37°C. Degranulation was represented as the fold increase of CD107a on NK cell surface, which is the difference between the percentage of NK cells expressing CD107a at surface after stimulation with P815 cells supplemented with agonist mAb, and the percentage of NK cells expressing CD107a at NK cell surface after incubation with P815 cells with no agonist mAb. NK cell degranulation assays were performed in 32 ALL patients and 10 age-matched controls. **(B)** Summary of data and statistic from 32 ALL patients and 10 age-matched controls. **(C)** Venn diagrams showing the number of functions as defined by Boolean gating. The diagrams show the concurrent expression of various NK cell receptors in those patients that displayed degranulation values under the 50th percentile.

An impaired NK cell effector functions has been associated with a higher incidence of cancer including hematological malignancies (48, 49). However, there are few studies assessing the functional competence of NK cells in pediatric patients with ALL. Our previous work (19) and that published by Rouce and collaborators (17) has provided information on the state of NK cell responsiveness at moment of diagnosis. Since we found that the expression of multiple receptors was significantly affected in NK cells from patients compared with healthy controls, we decide to assess NK cell-mediated cytotoxicity through an activating receptor whose expression was not significantly different between patients and healthy controls, as is the case for CD16, the low affinity receptor for IgG. Therefore, we analyzed NK cell degranulation in reverse ADCC assays by using P815 cells supplemented with anti-CD16 mAbs. Our results showed that the NK degranulation, measured as the expression of CD107a at NK cell surface, was significantly reduced when compared with healthy controls (**Figures 6A, B**). These results demonstrate that ADCC is also affected in NK cells from ALL pediatric patients. In addition, by Boolean analysis we evaluated the expression of various NK cell receptors in those patients that displayed an abnormal degranulation according to the percentile 50th value. Interestingly, we found that more than 50% of the patients with impaired degranulation displayed a concurrent decrease in the percentage of NK cells expressing three different type of receptors (**Figure 6C**).

### A decreased expression of activating receptors in NK cells is associated with acute lymphoblastic leukemia

By mean of a logistic regression analysis, we evaluated a possible association between an abnormal NK cell phenotype, highlighted by a significant decrease in the relative frequencies and a downregulated expression of various activating receptors, with the probability of developing leukemia. To perform such analysis, the categorical variables, expressed as the percentage of NK cells expressing a given activating receptor or, as mean fluorescence intensity (MFI) for each receptor, were adjusted within the model of logistic regression. When the analysis was adjusted by sex and age, we observed that a higher percentage of NK cells expressing NKG2D was associated with a lower probability of developing ALL (aOR= 0.23; 95% CI:0.08.-0.67). A similar trend was observed for DNAM-1 (aOR= 0.18; 95% CI: 0.06-0.56). In contrast to NKG2D, we also found that a higher expression density for DNAM-1 on NK cell surface was associated with lymphoblastic leukemia at the moment of diagnosis (aOR=0.30; 95% CI:0.10-0.89). Furthermore, our analysis showed that higher percentage of NK cells expressing 2B4, NTBA or SLAMF7, were also associated with a lower probability of developing ALL during childhood (**Table 2**). Finally, the logistic regression analysis also showed that an increase in the percentages of the two major NK cell subsets in peripheral blood (NK CD56^dim^ and NK CD56^bright^) was associated with a lower probability of developing the disease. However, no association was identified for the relative frequencies of NK cells subsets distinguished by the differential expression of NKG2A and CD57 (**Table 2**). When the logistic regression analysis was adjusted by sex, age and infection, we found that a decrease in the percentage of DNAM-1^+^NK cells, 2B4^+^NK cells, NTBA^+^NK cells, and SLAMF7^+^ NK cells, as well as a decrease in their expression levels on NK cells, were associated with lymphoblastic acute leukemia during childhood at the moment of ALL diagnosis (**Table 2**). Same results were obtained for the percentage of NK cells expressing NKG2D and percentages of CD56^dim^ NK cells and CD56 ^bright^ NK cells (**Table 2**). Notably, a decreased percentage of NKG2C+ NK cell was significantly associated with acute lymphoblastic leukemia when the categorical variable was adjusted by sex, age and allergies history (**Table 2**).

**Table 2:** Logistic regression analyses for the association between NK cells features (as categorical variables) and the risk of acute lymphoblastic leukemias in Mexican children.

NK cells features	OR	Sex and age	Sex, age and infections	Sex, age and allergies
		aOR (95% CI)	aOR (95% CI)	aOR (95% CI)
**MIF Immunoreceptors ≥p50 ^1^**				
NKG2D	0.47 (0.17-1.27)	0.42 (0.15-1.21)	0.43 (0.15-1.23)	0.51 (0.17-1.50)
NKG2A	1.65 (0.61-4.50)	1.64 (0.60-4.48)	1.60 (0.58-4.43)	1.82 (0.64-5.17)
NKG2C	0.54 (0.20-1.46)	0.53 (0.19-1.47)	0.52 (0.19-1.44)	0.45 (0.16-1.31)
NKP46	0.54 (0.20-1.46)	0.52 (0.19-1.44)	0.53 (0.19-1.45)	0.53 (0.19-1.50)
**CD69**	0.37 (0.13-1.03)	0.32 (0.11-0.95)*	0.29 (0.10-0.90)*	0.27 (0.09-0.85)*
TIGIT	0.50 (0.18-1.36)	0.46 (0.16-1.30)	0.46 (0.16-1.30)	0.54 (0.18-1.58)
CD96	0.40 (0.15-1.10)	0.38 (0.13-1.08)	0.38 (0.13-1.11)	0.42 (0.14-1.24)
**DNAM**	0.34 (0.12-0.95)*	0.30 (0.10-0.89)*	0.29 (0.10-0.86)*	0.31 (0.10-0.94)*
**CD57**	0.32 (0.11-0.88)*	0.31 (0.11-0.88)*	0.32 (0.11-0.89)*	0.34 (0.12-0.99)*
CD85j	0.54 (0.20-1.46)	0.48 (0.17-1.40)	0.49 (0.17-1.44)	0.41 (0.13-1.25)
**CRACC**	0.29 (0.10-0.82)*	0.28 (0.10-0.80)*	0.28 (0.10-0.79)*	0.31 (0.11-0.91)*
**2B4**	0.32 (0.11-0.88)*	0.30 (0.10-0.85)*	0.30 (0.10-0.85)*	0.33 (0.11-0.96)*
**NTBA**	0.26 (0.09-0.75)*	0.26 (0.09-0.75)*	0.27 (0.09-0.76)*	0.32 (0.11-0.96)*
CD16	0.80 (0.30-2.15)	0.80 (0.29-2.19)	0.81 (0.30-2.21)	0.99 (0.35-2.85)
**% NK positive for immunoreceptors ≥p50 ^2^**				
**NKG2D**	0.26 (0.09-0.72)*	0.23 (0.08-0.67)**	0.23 (0.08-0.67)**	0.26 (0.09-0.81)*
NKG2A	1.71 (0.64-4.57)	1.73 (0.64-4.66)	1.74 (0.64-4.70)	2.17 (0.76-6.22)
**NKG2C**	0.37 (0.14-1.01)	0.37 (0.13-1.00)	0.37 (0.13-1.01)	0.30 (0.10-0.88)*
NKP46	0.51 (0.19-1.37)	0.48 (0.17-1.34)	0.48 (0.17-1.33)	0.54 (0.19-1.55)
CD69	0.77 (0.29-2.04)	0.74 (0.27-2.05)	0.72 (0.26-2.03)	0.79 (0.28-2.24)
TIGIT	0.45 (0.17-1.22)	0.43 (0.16-1.18)	0.43 (0.16-1.19)	0.43 (0.15-1.22)
CD96	0.45 (0.17-1.22)	0.43 (0.16-1.18)	0.43 (0.16-1.18)	0.45 (0.16-1.27)
**DNAM**	0.22 (0.08-0.62)**	0.18 (0.06-0.56)**	0.18 (0.06-0.56)**	0.22 (0.07-0.72)*
CD57	0.43 (0.16-1.15)	0.43 (0.16-1.15)	0.43 (0.16-1.15)	0.45 (0.16-1.25)
CD85	0.86 (0.32-2.28)	0.82 (0.30-2.26)	0.82 (0.30-2.27)	0.77 (0.27-2.17)
**CRACC**	0.26 (0.09-0.72)*	0.25 (0.09-0.70)**	0.24 (0.08-0.68)**	0.25 (0.09-0.73)*
**2B4**	0.20 (0.07-0.57)**	0.13 (0.04-0.46)**	0.13 (0.04-0.46)**	0.14 (0.04-0.51)**
**NTBA**	0.22 (0.08-0.62)**	0.21 (0.07-0.60)**	0.21 (0.07-0.60)**	0.24 (0.08-0.73)*
CD16**^1^**	0.61 (0.23-1.66)	0.61 (0.23-1.67)	0.61 (0.23-1.67)	0.73 (0.26-2.07)
**% Subpopulations ≥p50**				
**NK Dim ^2^**	0.05 (0.01-0.20)***	0.05 (0.01-0.18)***	0.04 (0.01-0.16)***	0.04 (0.01-0.18)***
**NK Bright ^2^**	0.27 (0.09-0.75)*	0.25 (0.09-0.72)*	0.25 (0.09-0.72)*	0.28 (0.09-0.83)*
**NK Total ^2^**	0.10 (0.03-0.33)***	0.08 (0.02-0.29)***	0.07 (0.02-0.26)***	0.07 (0.02-0.29)***
NKG2A- CD57 ^1^	0.61 (0.23-1.66)	0.60 (0.22-1.64)	0.60 (0.22-1.65)	0.64 (0.23-1.80)
NKG2A+ CD57- **^1^**	1.54 (0.57-4.19)	1.53 (0.56-4.17)	1.56 (0.57-4.28)	1.49 (0.53-4.16)
NKG2A+ CD57+ **^1^**	1.03 (0.38-2.79)	1.03 (0.38-2.78)	1.03 (0.38-2.79)	1.13 (0.40-3.17)
NKG2A- CD57+ **^1^**	0.61 (0.23-1.66)	0.61 (0.22-1.67)	0.61 (0.22-1.68)	0.55 (0.19-1.57)

ALL: acute lymphoblastic leukemia; OR: odds ratio; aOR: adjusted odds ratio; MIF: medium intensity of fluorescence; CI: confidence interval; >p50: above 50^th^ percentile; NK: Natural killer. ^1^ 62 cases and 21 controls; ^2^ 72 cases and 21 controls. *:p<0.05; **: p <0.01; ***: p <0.001. Bold values represent the statistically significant results.

Finally, associations between immunoreceptors and clinical characteristics of patient with ALL were also explored. Lower percentages of CD69^+^ NK cells (OR=0.32; 95% CI:0.12-0.87) and 2B4^+^ NK cells (OR=0.20; 95% CI:0.04-0.09) from patients older than 10-year-old were associated with higher risk of developing leukemia. In addition, an association between a higher percentage of 2B4^+^ NK cells (OR=5.69; 95% CI:1.16-27.92) with a lower risk of developing leukemia was observed only for male patients. A similar result was obtained for a higher percentage of NK cells expressing DNAM-1 (OR=4.05; 95% CI:1.05-15.59), but only in patients suffering from allergies. No other associations were noticed (**Supplementary Table 2**).

## Discussion

A delicate balance between activation and inhibitory signals largely determines whether an NK cell will kill or not the target cell. Disruption of this equilibrium by altering the expression of activating and inhibitory receptors, results in an abnormal NK cell function that may contribute to cancer. Here, we analyzed the expression of various activating and inhibitory NK cell receptors in pediatric ALL patients at diagnosis.

The role of NCR receptors in the recognition and killing of cancer cells has been well documented (17, 50–53). Consistent with prior findings, we observed that the frequencies of NKp46^+^ NK cells at diagnosis were diminished in ALL patients compared to healthy controls. Our study did not involve the analysis of other members of the NCR family such as NKp44 or NKp30, however their expression were reported to be normal in pediatric patients with ALL (17). Since a distinct non-overlapping function has been attributed to these receptors (54), we cannot exclude the possibility of an abnormal expression for such receptors in our patients cohort. NKG2C is involved in the recognition of HLA-E molecules (55–57). In this study we found that the percentage of NKG2C+NK cells and overall expression of NKG2C on NK cells were significantly reduced in ALL patients. The recognition of HLA-E by NK cells may represent a mechanism for immunosurveillance of normal biosynthesis of HLA class I molecules, a process dampened in certain virus-infected or cancer cells including leukemia (58). Thus, our results suggest that NKG2C may play an important role in the recognition and killing of leukemic cells, especially when the dominant contribution of inhibitory receptors, such as NKG2A, falls beneath a critical threshold (58, 59). NKG2D is a NK cell receptor, which ligands represented by ULBPs and MICA/B are upregulated in cancers cells (60–62). In contrast to the study by Rouce and colleagues (17), we found a significant reduction in the percentage of NK cells expressing NKG2D in ALL patients when compared with healthy controls. A significant downregulation in the expression of NKG2D on NK cell surface was also noticed. A possible explanation to this discrepancy may be due to differences in the percentages of patients diagnosed with low and high risk ALL. In our study, 37 (51.4%) patients were diagnosed with high risk leukemia compared with the 32% reported by Rouce. However, further studies will be useful to precise these findings. DNAM-1 is an activating receptor that upon ligation with PVR and Nectin-2, triggers NK cell-mediated cytotoxicity against various cancer cells. Here, we noticed a significant reduction in the percentage of DNAM+ NK cells and overall expression of DNAM. Our data support the notion that DNAM-1 plays an important role in the recognition and killing of leukemic cells. Because TIGIT competes with DNAM-1 for binding to PVR (CD155), a downregulation of DNAM-1 in NK cells may favor inhibitory signaling, even if the expression levels of TIGIT remain relatively unchanged, as observed in our patient cohort (63, 64). By contrast, CD96 also represents a competing receptor for PVR, which expression pattern in lymphoid cells is similar but not identical to TIGIT (65–67). Unlike TIGIT, we observed that the percentage of CD96^+^ NK cells and its overall expression were significant reduced in patients with ALL. Whereas the functional relevance of CD96 ligation by PVR needs to be determined, our results suggest that CD96 may play an important role in the recognition of ALL blast and contribute to modulate the delicate balance stablished between the DNAM-1-TIGIT-CD96 axis. The expression of SLAM family receptors on hematopoietic targets cells make them highly sensitive to NK cell-mediated cytotoxicity when compared with targets cells lacking expression of SLAM receptors (68, 69). We found that the proportions of NK cell expressing 2B4, NTBA or SLAM were all significantly decreased in ALL patients when compared with healthy controls. Moreover, a downregulation in the expression of these SLAM receptors was also observed. To our knowledge, this is the first study documenting an aberrant expression of SLAM family receptors in pediatric patients with ALL.

Boolean analysis showed that a significant majority of ALL patients displayed a concurrent decrease in the expression percentage for at least two different type of receptors. Moreover, same studies demonstrated that among ALL patients, NK cells with a concurrent normal expression for more than one single type of receptor is the less common phenotype. To our knowledge, this is first report showing an abnormal NK cell phenotype highlighted by the concurrent decreased expression in at least more than one single type of activating receptor in children with ALL at diagnosis. Whether these phenotypes could be associated not only with the initial presentation of ALL but also with the prognosis of the disease should be investigated in future studies.

The gradual loss of NKG2A expression and the sequential acquisition of CD57 has been associated to different differentiation stages (19–22). We observed that the percentages of NKG2A^+^CD57^-^ NK cells were significant increase in patients with ALL when compared to healthy controls, whereas a significant decreased in the percentages of NKG2A^-^CD57^+^ NK cells was also observed. Our data suggest that lymphoblastic leukemia may impact on different aspects of NK cell biology including differentiation.

A failure in the NK cell-mediated natural cytotoxicity has been shown in pediatric patients with ALL (17, 19), however, the molecular mechanism behind this abnormal effector function remain elusive. Since an abnormal expression of activating and inhibitory receptors has been also documented in ALL, a possible explanation for the impaired NK cell cytotoxicity in such patients, may be the consequence of an altered balance of activating or inhibitory signaling. In order to bypass the influence of this abnormal expression of activation receptors on NK cell function, we performed NK cell degranulation assays in response to an activating receptor whose expression on NK cell surface remain unaltered between patients and healthy controls as turn to be the case for CD16. Our results showed than despite a normal expression of CD16 in NK cells from ALL patients, we observed a significant reduction of in the ability of NK cells to degranulate in response to P815 cells supplemented with anti-CD16. Moreover, more than 50 percent of those patients with a poor ability to degranulate also displayed a simultaneous decreased in the percentage of NK cells expressing various activating receptors. These results suggest than in addition to an abnormal expression of activating receptors, other factors intrinsic to the cytolytic machinery may be also compromised in NK cell from pediatric patients with ALL.

A logistic regression analysis was conducted to identify associations between an altered phenotype of NK cells and the probability of developing acute lymphoblastic leukemia during childhood. Our analysis indicates that there is a significant association between a reduction, either in the percentage or density expression of a determined NK cell receptor and lymphoblastic leukemia during childhood at moment of diagnosis. This association was found for the activating receptors NKp46, NKG2C, NKG2D, DNAM-1, 2B4, NTBA, and SLAMF7. Our results confirm that a downregulation in the expression of activating NK cell receptors may contribute with an inefficient killing of leukemic cells, a phenomenon also observed in other hematological malignancies including acute myeloid leukemia, where a diminished expression of NCRs, DNAM and NKG2D has been suggested to explain the resistance of myeloid leukemias to NK-mediated lysis (70).

A conclusion from this study is that a significant reduction in the expression of activating receptors was not limited to only one single type of receptor or family of activating receptors. Instead, we observed a concurrent downregulation of multiple types of receptors belonging to different families. Moreover, our data also suggest that an overall reduction of percentages of NK cells expressing a specific type of activating receptor and its downregulation is significant associated to acute lymphoblastic leukemia during childhood. Therefore, a normal expression of activating receptors in NK cells may be important to display an efficient cytotoxicity towards lymphoblastic acute leukemia.

## Data availability statement

The raw data supporting the conclusions of this article will be made available by the authors, without undue reservation.

## Author contributions

Contribution: LV-V and JCN-E performed research, performed statistical analysis, analyzed and interpreted data; JS-H performed research and performed statistical analysis; AM-S, MLP-S, EJ-H, JAM-T, MAC-M, JF-L, RA-S, FGM-R, JGP-G, DAD-R, JRT-N, RME-E, BC-H, LVF-V, LEM-P, CA-H, RR-C, KAS-L, FM-L, JAP-G, MMV-A, AM-R, AA-D, JDS-J, AG-S, AJG-V, MM-R, GAH-E, OAS-R, and HR-V collected data; IM-H analyzed and interpreted data; SJ-M and AH-M collected data and performed statistical analysis; IM-D, JDW, KWH, and KPM provided critical review and assisted in writing the manuscript; JMM-A and MEC-M analyzed and interpreted data, wrote the manuscript and designed research. All authors contributed to the article and approved the submitted version.

## Funding

MEC-M is supported by Consejo Nacional de Ciencia y Tecnología (FORDECYT-PRONACES-377883-2020). JMM-A is supported by FONCICYT/37/2018, FIS/IMSS/PROT/1782, and CB 2015-258042-M. JN-E is supported by FORDECYT/303019/2019.

## Acknowledgments

The authors want to acknowledge to Erika Melchy-Perez for her technical assistance in Flow Cytometry.

## Conflict of interest

Authors JDW, KWH, and KPM are employed by GlaxoSmithKline.

The remaining authors declare that the research was conducted in the absence of any commercial or financial relationships that could be construed as a potential conflict of interest.

## Publisher’s note

All claims expressed in this article are solely those of the authors and do not necessarily represent those of their affiliated organizations, or those of the publisher, the editors and the reviewers. Any product that may be evaluated in this article, or claim that may be made by its manufacturer, is not guaranteed or endorsed by the publisher.

## Supplementary material

Supplementary Figure 1 Percentages of NK cells CD56 dim and CD56 bright in healthy controls and ALL patients. **(A)** The percentages of total NK cells and NK cells CD56 dim were analyzed by flow cytometry in 21 healthy controls and 71 ALL patients. **(B)** The percentages of NK cells CD56 bright were analyzed by flow cytometry in 21 healthy controls and 71 ALL patients. Vertical lines indicate standard deviation. The horizonal lines represents the mean value. P values reports significance according to U-Mann Whitney Test one-tail. HC: healthy controls; ALL: Acute lymphoblastic leukemia patients.

Supplementary Figure 2 Percentages of CD16^+^; CD69^+^; CD57^+^ NK cells in healthy controls and ALL patients. **(A)** The frequencies of CD3^-^CD20^-^CD14^-^CD56^+^ cells expressing CD16, CD69 or, CD57 were analyzed by flow cytometry in 72 ALL patients and 21 healthy controls. Vertical lines indicate standard deviation. The horizonal lines represents the mean value. P values reports significance according to U-Mann Whitney Test one-tail. HC: healthy controls; ALL: Acute lymphoblastic leukemia patients. **(B)** Representative FACS plots depict the percentages of NK cells expressing CD16, CD69 or, CD57 from healthy controls (*n*=21) and ALL patients (*n*=72). HC: healthy controls; ALL: Acute lymphoblastic leukemia patients.

## References

[1] Greaves M. A causal mechanism for childhood acute lymphoblastic leukaemia. Nat Rev Cancer (2018) 18(8):471–84. doi: 10.1038/s41568-018-0015-6 PMC6986894 29784935

[2] Dunn GP Bruce AT Ikeda H Old LJ Schreiber RD. Cancer immunoediting: from immunosurveillance to tumor escape. Nat Immunol (2002) 3(11):991–8. doi: 10.1038/ni1102-991 12407406

[3] Dunn GP Old LJ Schreiber RD. The immunobiology of cancer immunosurveillance and immunoediting. Immunity (2004) 21(2):137–48. doi: 10.1016/j.immuni.2004.07.017 15308095

[4] Pastorczak A Domka K Fidyt K Poprzeczko M Firczuk M. Mechanisms of immune evasion in acute lymphoblastic leukemia. Cancers (Basel) (2021) 13(7):1–25. doi: 10.3390/cancers13071536 PMC8037152 33810515

[5] Chiossone L Dumas PY Vienne M Vivier E. Natural killer cells and other innate lymphoid cells in cancer. Nat Rev Immunol (2018) 18(11):671–88. doi: 10.1038/s41577-018-0061-z 30209347

[6] Pende D Marcenaro S Falco M Martini S Bernardo ME Montagna D. Anti-leukemia activity of alloreactive NK cells in KIR ligand-mismatched haploidentical HSCT for pediatric patients: Evaluation of the functional role of activating KIR and redefinition of inhibitory KIR specificity. Blood (2009) 113(13):3119–29. doi: 10.1182/blood-2008-06-164103 18945967

[7] Locatelli F Pende D Maccario R Mingari MC Moretta A Moretta L. Haploidentical hemopoietic stem cell transplantation for the treatment of high-risk leukemias: how NK cells make the difference. Clin Immunol (2009) 133(2):171–8. doi: 10.1016/j.clim.2009.04.009 19481979

[8] Locatelli F Pende D Mingari MC Bertaina A Falco M Moretta A. Cellular and molecular basis of haploidentical hematopoietic stem cell transplantation in the successful treatment of high-risk leukemias: Role of alloreactive NK cells. Front Immunol (2013) 4:15. doi: 10.3389/fimmu.2013.00015 23378843 PMC3561663

[9] Velardi A Ruggeri L Alessandro Moretta Moretta L. NK cells: a lesson from mismatched hematopoietic transplantation. Trends Immunol (2002) 23(9):438–44. doi: 10.1016/S1471-4906(02)02284-6 12200065

[10] Fehniger TA Cooper MA. Harnessing NK cell memory for cancer immunotherapy. Trends Immunol (2016) 37(12):877–88. doi: 10.1016/j.it.2016.09.005 PMC5135622 27773685

[11] Bi J Tian Z. NK cell dysfunction and checkpoint immunotherapy. Front Immunol (2019) 10:1999. doi: 10.3389/fimmu.2019.01999 31552017 PMC6736636

[12] Daher M Rezvani K. The evolution of NK cell immunotherapy for hematologic malignancies: A historical and contemporary perspective. Semin Hematol (2020) 57(4):165–6. doi: 10.1053/j.seminhematol.2020.11.005 PMC9896580 33256908

[13] Waldhauer I Steinle A. NK cells and cancer immunosurveillance. Oncogene (2008) 27(45):5932–43. doi: 10.1038/onc.2008.267 18836474

[14] Romanski A Bug G Becker S Kampfmann M Seifried E Hoelzer D. Mechanisms of resistance to natural killer cell-mediated cytotoxicity in acute lymphoblastic leukemia. Exp Hematol (2005) 33(3):344–52. doi: 10.1016/j.exphem.2004.11.006 15730858

[15] Reusing SB Manser AR Enczmann J Mulder A Claas FH Carrington M. Selective downregulation of HLA-c and HLA-e in childhood acute lymphoblastic leukaemia. Br J Haematol (2016) 174(3):477–80. doi: 10.1111/bjh.13777 PMC4854806 26527563

[16] Makanga DR Da Rin de Lorenzo F David G Willem C Dubreuil L Legrand N. Genetic and molecular basis of heterogeneous NK cell responses against acute leukemia. Cancers (Basel) (2020) 12(7):1–18. doi: 10.3390/cancers12071927 PMC7409189 32708751

[17] Rouce RH Shaim H Sekine T Weber G Ballard B Ku S. The TGF-beta/SMAD pathway is an important mechanism for NK cell immune evasion in childhood b-acute lymphoblastic leukemia. Leukemia (2016) 30(4):800–11. doi: 10.1038/leu.2015.327 PMC4823160 26621337

[18] Parrado A Casares S Rodriguez-Fernandez JM. Natural killer cytotoxicity and lymphocyte subpopulations in patients with acute leukemia. Leuk Res (1994) 18(3):191–7. doi: 10.1016/0145-2126(94)90114-7 7511191

[19] Valenzuela-Vazquez L Nunez-Enriquez JC Sanchez-Herrera J Jimenez-Hernandez E Martin-Trejo JA Espinoza-Hernandez LE. Functional characterization of NK cells in Mexican pediatric patients with acute lymphoblastic leukemia: Report from the Mexican interinstitutional group for the identification of the causes of childhood leukemia. PloS One (2020) 15(1):e0227314. doi: 10.1371/journal.pone.0227314 31951638 PMC6968843

[20] Liu S Dhar P Wu JD. NK cell plasticity in cancer. J Clin Med (2019) 8(9):1–17. doi: 10.3390/jcm8091492 PMC6780970 31546818

[21] Lanier LL. NK cell recognition. Annu Rev Immunol (2005) 23:225–74. doi: 10.1146/annurev.immunol.23.021704.115526 15771571

[22] Long EO Kim HS Liu D Peterson ME Rajagopalan S. Controlling natural killer cell responses: integration of signals for activation and inhibition. Annu Rev Immunol (2013) 31:227–58. doi: 10.1146/annurev-immunol-020711-075005 PMC3868343 23516982

[23] Giuliani M Poggi A. Checkpoint inhibitors and engineered cells: New weapons for natural killer cell arsenal against hematological malignancies. Cells (2020) 9(7). doi: 10.3390/cells9071578 PMC7407972 32610578

[24] Sivori S Pende D Bottino C Marcenaro E Pessino A Biassoni R. NKp46 is the major triggering receptor involved in the natural cytotoxicity of fresh or cultured human NK cells. correlation between surface density of NKp46 and natural cytotoxicity against autologous, allogeneic or xenogeneic target cells. Eur J Immunol (1999) 29(5):1656–66. 10.1002/(SICI)1521-4141(199905)29:05<1656::AID-IMMU1656>3.0.CO;2-1 10359120

[25] Thielens A Vivier E Romagne F. NK cell MHC class I specific receptors (KIR): from biology to clinical intervention. Curr Opin Immunol (2012) 24(2):239–45. doi: 10.1016/j.coi.2012.01.001 22264929

[26] Parham P. MHC class I molecules and KIRs in human history, health and survival. Nat Rev Immunol (2005) 5(3):201–14. doi: 10.1038/nri1570 15719024

[27] Muntasell A Ochoa MC Cordeiro L Berraondo P Lopez-Diaz de Cerio A Cabo M. Targeting NK-cell checkpoints for cancer immunotherapy. Curr Opin Immunol (2017) 45:73–81. doi: 10.1016/j.coi.2017.01.003 28236750

[28] Konjevic G Jurisic V Jovic V Vuletic A Mirjacic Martinovic K Radenkovic S. Investigation of NK cell function and their modulation in different malignancies. Immunol Res (2012) 52(1-2):139–56. doi: 10.1007/s12026-012-8285-7 22442005

[29] Molgora M Cortez VS Colonna M. Killing the invaders: NK cell impact in tumors and anti-tumor therapy. Cancers (Basel) (2021) 13(4). doi: 10.3390/cancers13040595 PMC7913353 33546248

[30] Costello RT Sivori S Marcenaro E Lafage-Pochitaloff M Mozziconacci MJ Reviron D. Defective expression and function of natural killer cell-triggering receptors in patients with acute myeloid leukemia. Blood (2002) 99(10):3661–7. doi: 10.1182/blood.V99.10.3661 11986221

[31] Costello RT Fauriat C Sivori S Marcenaro E Olive D. NK cells: innate immunity against hematological malignancies? Trends Immunol (2004) 25(6):328–33. doi: 10.1016/j.it.2004.04.005 15145323

[32] Sanchez CJ Le Treut T Boehrer A Knoblauch B Imbert J Olive D. Natural killer cells and malignant haemopathies: a model for the interaction of cancer with innate immunity. Cancer Immunol Immunother (2011) 60(1):1–13. doi: 10.1007/s00262-010-0898-x 20697893 PMC11029698

[33] Viel S Charrier E Marcais A Rouzaire P Bienvenu J Karlin L. Monitoring NK cell activity in patients with hematological malignancies. Oncoimmunology (2013) 2(9):e26011. doi: 10.4161/onci.26011 24327939 PMC3850490

[34] Zhang C Liu Y. Targeting NK cell checkpoint receptors or molecules for cancer immunotherapy. Front Immunol (2020) 11:1295. doi: 10.3389/fimmu.2020.01295 32714324 PMC7344328

[35] Rudant J Lightfoot T Urayama KY Petridou E Dockerty JD Magnani C. Childhood acute lymphoblastic leukemia and indicators of early immune stimulation: a childhood leukemia international consortium study. Am J Epidemiol (2015) 181(8):549–62. doi: 10.1093/aje/kwu298 PMC4850899 25731888

[36] Nunez-Enriquez JC Fajardo-Gutierrez A Buchan-Duran EP Bernaldez-Rios R Medina-Sanson A Jimenez-Hernandez E. Allergy and acute leukaemia in children with down syndrome: a population study. report from the Mexican inter-institutional group for the identification of the causes of childhood leukaemia. Br J Cancer (2013) 108(11):2334–8. 10.1038/bjc.2013.237 PMC3681010 23695017

[37] Bryceson YT Fauriat C Nunes JM Wood SM Bjorkstrom NK Long EO. Functional analysis of human NK cells by flow cytometry. Methods Mol Biol (2010) 612:335–52. doi: 10.1007/978-1-60761-362-6_23 PMC4969010 20033652

[38] Bryceson YT Pende D Maul-Pavicic A Gilmour KC Ufheil H Vraetz T. A prospective evaluation of degranulation assays in the rapid diagnosis of familial hemophagocytic syndromes. Blood (2012) 119(12):2754–63. doi: 10.1182/blood-2011-08-374199 22294731

[39] Arellano-Galindo J Barrera AP Jimenez-Hernandez E Zavala-Vega S Campos-Valdez G Xicohtencatl-Cortes J. Infectious agents in childhood leukemia. Arch Med Res (2017) 48(4):305–13. doi: 10.1016/j.arcmed.2017.09.001 29157671

[40] Brenchley JM Karandikar NJ Betts MR Ambrozak DR Hill BJ Crotty LE. Expression of CD57 defines replicative senescence and antigen-induced apoptotic death of CD8+ T cells. Blood (2003) 101(7):2711–20. doi: 10.1182/blood-2002-07-2103 12433688

[41] Chattopadhyay PK Betts MR Price DA Gostick E Horton H Roederer M. The cytolytic enzymes granyzme a, granzyme b, and perforin: expression patterns, cell distribution, and their relationship to cell maturity and bright CD57 expression. J Leukoc Biol (2009) 85(1):88–97. 18820174 10.1189/jlb.0208107 PMC2638730

[42] Lopez-Verges S Milush JM Pandey S York VA Arakawa-Hoyt J Pircher H. CD57 defines a functionally distinct population of mature NK cells in the human CD56dimCD16+ NK-cell subset. Blood (2010) 116(19):3865–74. doi: 10.1182/blood-2010-04-282301 PMC2981540 20733159

[43] Boles KS Vermi W Facchetti F Fuchs A Wilson TJ Diacovo TG. A novel molecular interaction for the adhesion of follicular CD4 T cells to follicular DC. Eur J Immunol (2009) 39(3):695–703. doi: 10.1002/eji.200839116 19197944 PMC3544471

[44] Stanietsky N Simic H Arapovic J Toporik A Levy O Novik A. The interaction of TIGIT with PVR and PVRL2 inhibits human NK cell cytotoxicity. Proc Natl Acad Sci U S A (2009) 106(42):17858–63. doi: 10.1073/pnas.0903474106 PMC2764881 19815499

[45] Joller N Hafler JP Brynedal B Kassam N Spoerl S Levin SD. Cutting edge: TIGIT has T cell-intrinsic inhibitory functions. J Immunol (2011) 186(3):1338–42. doi: 10.4049/jimmunol.1003081 PMC3128994 21199897

[46] Sanchez-Correa B Valhondo I Hassouneh F Lopez-Sejas N Pera A Bergua JM. DNAM-1 and the TIGIT/PVRIG/TACTILE axis: Novel immune checkpoints for natural killer cell-based cancer immunotherapy. Cancers (Basel) (2019) 11(6):1–15. doi: 10.3390/cancers11060877 PMC6628015 31234588

[47] Colonna M Nakajima H Navarro F Lopez-Botet M. A novel family of ig-like receptors for HLA class I molecules that modulate function of lymphoid and myeloid cells. J Leukoc Biol (1999) 66(3):375–81. doi: 10.1002/jlb.66.3.375 10496306

[48] Finn OJ. Immuno-oncology: understanding the function and dysfunction of the immune system in cancer. Ann Oncol (2012) 23 Suppl 8:viii6–9. doi: 10.1093/annonc/mds256 PMC4085883 22918931

[49] Baier C Fino A Sanchez C Farnault L Rihet P Kahn-Perles B. Natural killer cells modulation in hematological malignancies. Front Immunol (2013) 4:459. doi: 10.3389/fimmu.2013.00459 24391641 PMC3867693

[50] Moretta A Biassoni R Bottino C Mingari MC Moretta L. Natural cytotoxicity receptors that trigger human NK-cell-mediated cytolysis. Immunol Today (2000) 21(5):228–34. doi: 10.1016/S0167-5699(00)01596-6 10782054

[51] Pessino A Sivori S Bottino C Malaspina A Morelli L Moretta L. Molecular cloning of NKp46: a novel member of the immunoglobulin superfamily involved in triggering of natural cytotoxicity. J Exp Med (1998) 188(5):953–60. doi: 10.1084/jem.188.5.953 PMC3207313 9730896

[52] Stringaris K Sekine T Khoder A Alsuliman A Razzaghi B Sargeant R. Leukemia-induced phenotypic and functional defects in natural killer cells predict failure to achieve remission in acute myeloid leukemia. Haematologica (2014) 99(5):836–47. doi: 10.3324/haematol.2013.087536 PMC4008119 24488563

[53] Montaldo E Del Zotto G Della Chiesa M Mingari MC Moretta A De Maria A. Human NK cell receptors/markers: a tool to analyze NK cell development, subsets and function. Cytometry A (2013) 83(8):702–13. doi: 10.1002/cyto.a.22302 23650273

[54] El Costa H Casemayou A Aguerre-Girr M Rabot M Berrebi A Parant O. Critical and differential roles of NKp46- and NKp30-activating receptors expressed by uterine NK cells in early pregnancy. J Immunol (2008) 181(5):3009–17. doi: 10.4049/jimmunol.181.5.3009 18713971

[55] Braud VM Allan DS O'Callaghan CA Soderstrom K D'Andrea A Ogg GS. HLA-e binds to natural killer cell receptors CD94/NKG2A, b and c. Nature (1998) 391(6669):795–9. doi: 10.1038/35869 9486650

[56] Borrego F Ulbrecht M Weiss EH Coligan JE Brooks AG. Recognition of human histocompatibility leukocyte antigen (HLA)-e complexed with HLA class I signal sequence-derived peptides by CD94/NKG2 confers protection from natural killer cell-mediated lysis. J Exp Med (1998) 187(5):813–8. doi: 10.1084/jem.187.5.813 PMC2212178 9480992

[57] Lopez-Botet M Muntasell A Vilches C. The CD94/NKG2C+ NK-cell subset on the edge of innate and adaptive immunity to human cytomegalovirus infection. Semin Immunol (2014) 26(2):145–51. doi: 10.1016/j.smim.2014.03.002 24666761

[58] Lopez-Botet M Bellon T Llano M Navarro F Garcia P de Miguel M. Paired inhibitory and triggering NK cell receptors for HLA class I molecules. Hum Immunol (2000) 61(1):7–17. doi: 10.1016/S0198-8859(99)00161-5 10658973

[59] Morandi F Pistoia V. Inter actions between HLA-G and HLA-e in physiological and pathological conditions. Front Immunol (2014) 5:394. doi: 10.3389/fimmu.2014.00394 25202308 PMC4141331

[60] Bauer S Groh V Wu J Steinle A Phillips JH Lanier LL. Activation of NK cells and T cells by NKG2D, a receptor for stress-inducible MICA. Science (1999) 285(5428):727–9. doi: 10.1126/science.285.5428.727 10426993

[61] Raulet DH Gasser S Gowen BG Deng W Jung H. Regulation of ligands for the NKG2D activating receptor. Annu Rev Immunol (2013) 31:413–41. doi: 10.1146/annurev-immunol-032712-095951 PMC4244079 23298206

[62] Lanier LL. NKG2D receptor and its ligands in host defense. Cancer Immunol Res (2015) 3(6):575–82. doi: 10.1158/2326-6066.CIR-15-0098 PMC4457299 26041808

[63] Johnston RJ Comps-Agrar L Hackney J Yu X Huseni M Yang Y. The immunoreceptor TIGIT regulates antitumor and antiviral CD8(+) T cell effector function. Cancer Cell (2014) 26(6):923–37. doi: 10.1016/j.ccell.2014.10.018 25465800

[64] Chauvin JM Ka M Pagliano O Menna C Ding Q DeBlasio R. IL15 stimulation with TIGIT blockade reverses CD155-mediated NK-cell dysfunction in melanoma. Clin Cancer Res (2020) 26(20):5520–33. doi: 10.1158/1078-0432.CCR-20-0575 PMC8045409 32591463

[65] Wang PL O'Farrell S Clayberger C Krensky AM. Identification and molecular cloning of tactile. a novel human T cell activation antigen that is a member of the ig gene superfamily. J Immunol (1992) 148(8):2600–8. 1313846

[66] Fuchs A Cella M Giurisato E Shaw AS Colonna M. Cutting edge: CD96 (tactile) promotes NK cell-target cell adhesion by interacting with the poliovirus receptor (CD155). J Immunol (2004) 172(7):3994–8. doi: 10.4049/jimmunol.172.7.3994 15034010

[67] Lepletier A Lutzky VP Mittal D Stannard K Watkins TS Ratnatunga CN. The immune checkpoint CD96 defines a distinct lymphocyte phenotype and is highly expressed on tumor-infiltrating T cells. Immunol Cell Biol (2019) 97(2):152–64. doi: 10.1111/imcb.12205 30222899

[68] Cruz-Munoz ME Valenzuela-Vazquez L Sanchez-Herrera J Santa-Olalla Tapia J. From the "missing self" hypothesis to adaptive NK cells: Insights of NK cell-mediated effector functions in immune surveillance. J Leukoc Biol (2019) 105(5):955–71. doi: 10.1002/JLB.MR0618-224RR 30848847

[69] Dong Z Cruz-Munoz ME Zhong MC Chen R Latour S Veillette A. Essential function for SAP family adaptors in the surveillance of hematopoietic cells by natural killer cells. Nat Immunol (2009) 10(9):973–80. doi: 10.1038/ni.1763 19648922

[70] Farnault L Sanchez C Baier C Le Treut T Costello RT. Hematological malignancies escape from NK cell innate immune surveillance: mechanisms and therapeutic implications. Clin Dev Immunol (2012) 2012:421702. doi: 10.1155/2012/421702 22899948 PMC3415262

