# Supplementary figures and images for: NK cells with decreased expression of multiple activating receptors is a dominant phenotype in pediatric patients with acute lymphoblastic leukemia
Source: Front Oncol. 2022 Nov 7;12:1023510. doi: 10.3389/fonc.2022.1023510 (PMC9677112; doi:10.3389/fonc.2022.1023510)

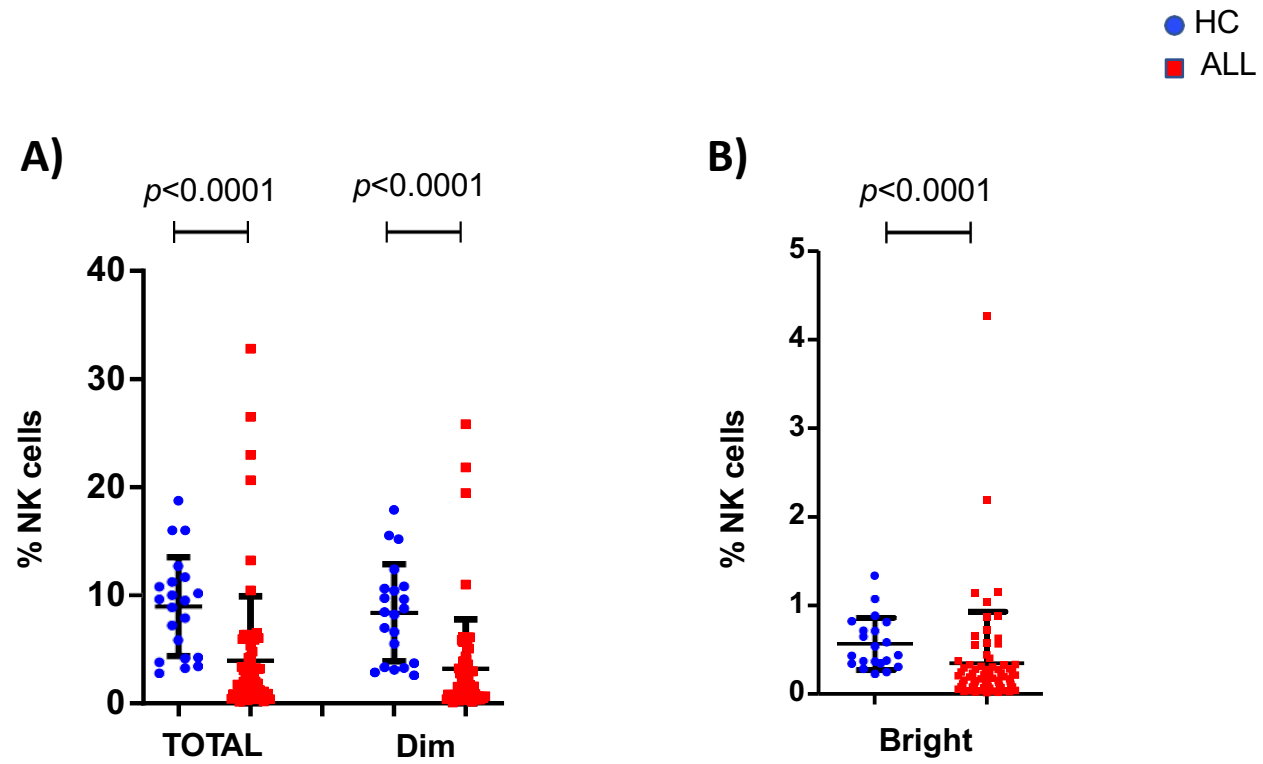

Supplementary Figure 1

A)

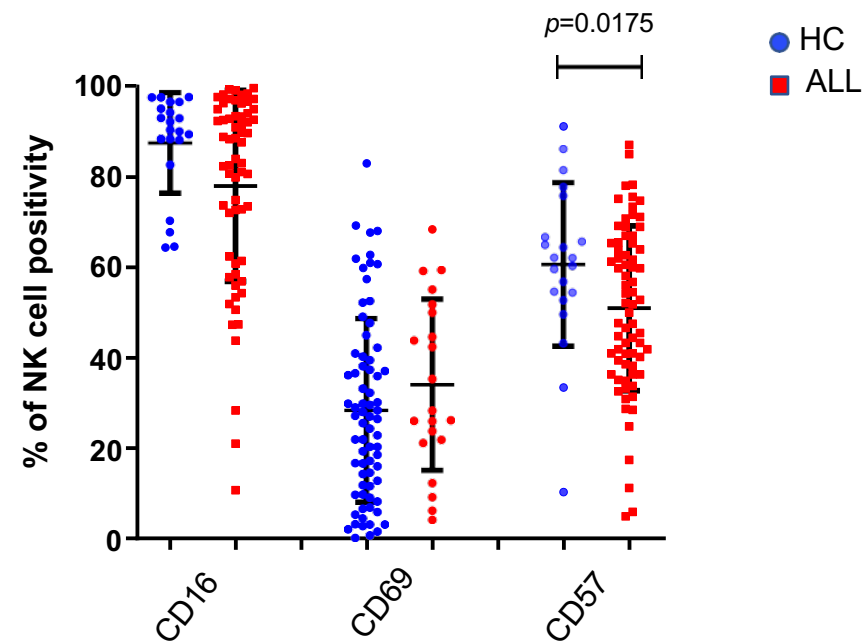

B)

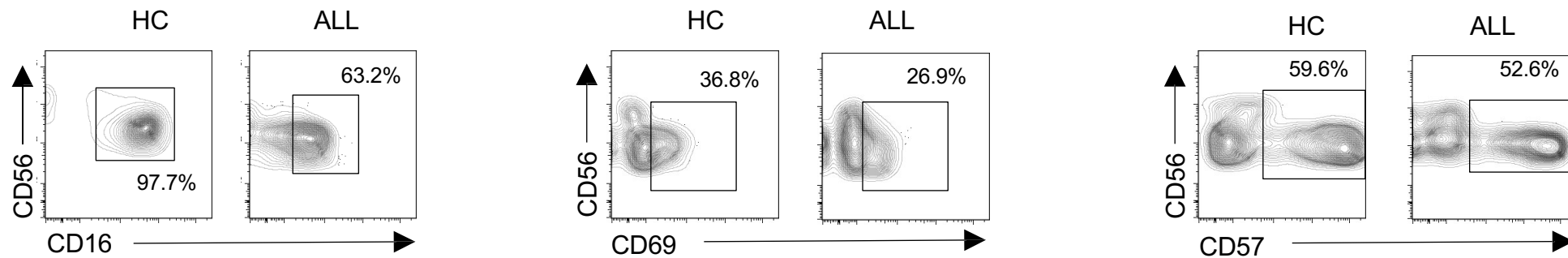

Supplement: Supplementary Figure 1 — Percentages of NK cells CD56 dim and CD56 bright in healthy controls and ALL patients. (A) The percentages of total NK cells and NK cells CD56 dim were analyzed by flow cytometry in 21 healthy controls and 71 ALL patients. (B) The percentages of NK cells CD56 bright were analyzed by flow cytometry in 21 healthy controls and 71 ALL patients. Vertical lines indicate standard deviation. The horizonal lines represents the mean value. P values reports significance according to U-Mann Whitney Test one-tail. HC: healthy controls; ALL: Acute lymphoblastic leukemia patients. [file DataSheet_1.pdf]
