# Supplementary material for: NK cells with decreased expression of multiple activating receptors is a dominant phenotype in pediatric patients with acute lymphoblastic leukemia
Source: Front Oncol. 2022 Nov 7;12:1023510. doi: 10.3389/fonc.2022.1023510 (PMC9677112; doi:10.3389/fonc.2022.1023510)
Supplement: Supplementary file 2 [file DataSheet_2.docx]

**Supplementary Table 1.** Immunoreceptors evaluated in NK cells.

| **Tube #** | **Antibody** | **Clone** | **Source** |
| --- | --- | --- | --- |
| 1 | FITC -Conjugated anti-CD3 | OKT3 | Biolegend |
|  | FITC -Conjugated anti-CD14 | 63D3 | Biolegend |
|  | FITC -Conjugated anti-CD20 | 2H7 | Biolegend |
|  | APC -Conjugated anti-CD56 | 5.1H11 | Biolegend |
|  | PerCP / Cy5.5-Conjugated anti-NKG2D | 1D11 | Biolegend |
|  | PE -Conjugated anti-NKG2A | REA110 | Miltenyi |
|  | PE / Vio 770-Conjugated anti-NKG2C | REA205 | Miltenyi |
|  | Pacific Blue -Conjugated anti-CD57 | HCD57 | Biolegend |
|  | Brilliant Violet 510-Conjugated anti-CD69 | FN50 | Biolegend |
|  | APC / Cy7-Conjugated anti-CD16 | 3G8 | Biolegend |
| 2 | FITC -Conjugated anti-CD3 | OKT3 | Biolegend |
|  | FITC -Conjugated anti-CD14 | 63D3 | Biolegend |
|  | FITC -Conjugated anti-CD20 | 2H7 | Biolegend |
|  | APC -Conjugated anti-CD56 | 5.1H11 | Biolegend |
|  | PerCP / Cy5.5-Conjugated anti-TIGIT | A15153G | Biolegend |
|  | PE -Conjugated anti-CD96 | NK92.39 | Biolegend |
|  | Brilliant Violet 510-Conjugated anti-DNAM | 11A8 | Biolegend |
|  | Pacific Blue -Conjugated anti-NKP46 | 9E2 | Biolegend |
| 3 | FITC -Conjugated anti-CD3 | OKT3 | Biolegend |
|  | FITC -Conjugated anti-CD14 | 63D3 | Biolegend |
|  | FITC -Conjugated anti-CD20 | 2H7 | Biolegend |
|  | APC -Conjugated anti-CD56 | 5.1H11 | Biolegend |
|  | PerCP / Cy5.5-Conjugated anti-CRACC | 162.1 | Biolegend |
|  | Pacific Blue -Conjugated anti-2B4 | C1.7 | Biolegend |
|  | PE / Vio 770-Conjugated anti-NTB-A | REA339 | Miltenyi |
|  | PE -Conjugated anti-CD85j | GHI/75 | Biolegend |

**Suplemmentary Table 2**

Association between NK cells features and clinical variables of pediatric patients with acute lymphoblastic leukemia (n=72).

| **NK cells features** | **Age >= 10**  *n=*29  OR (IC 95%) | **Sex Male**  *n=*40  OR (IC 95%) | **Infection during the first year of life**  **n=46**  OR (IC 95%) | **Allergy history**  **n=12**  OR (IC 95%) | **Leukocyte count >=50,000**  *n=*14  OR (IC 95%) | **Rearrangement**  *n=*5  OR (IC 95%) | **Immunophenotype T cell**  *n=*2  OR (IC 95%) | **Relapse**  *n=7*  OR (IC 95%) | **Death**  *n=8*  OR (IC 95%) |
| --- | --- | --- | --- | --- | --- | --- | --- | --- | --- |
| **MIF Immunoreceptors** **≥p50** | | | | | | | | | |
| NKG2D | 0.51 (0.16-1.58) | 1.90 (0.64-5.69) | 0.67 (0.22-2.04) | 2.25 (0.57-8.87) | 1.52 (0.42-5.53) | 0.97 (0.08-11.41) | NC | 1.33 (0.20-8.67) | 0.36 (0.04-3.30) |
| NKG2A | 0.87 (0.30-2.51) | 1.13 (0.40-3.21) | 1.33 (0.44-4.01) | 0.79 (0.20-3.18) | 0.47 (0.13-1.69) | 1.10 (0.09-12.92) | 0.54 (0.03-9.05) | 0.81 (0.12-5.26) | 0.24 (0.04-1.41) |
| NKG2C | 1.83 (0.64-5.26) | 1.33 (0.47-3.80) | 1.59 (0.51-4.95) | 0.37 (0.07-1.91) | 0.28 (0.05-1.39) | 0.84 (0.07-9.82) | NC | 0.40 (0.04-3.79) | 0.31 (0.03-2.83) |
| NKP46 | 1.03 (0.36-2.96) | 0.76 (0.27-2.14) | 1.14 (0.38-3.47) | 1.89 (0.48-7.40) | 0.28 (0.05-1.39) | 0.84 (0.07-9.82) | 1.73 (0.10-29.01) | 0.40 (0.04-3.79) | 0.31 (0.03-2.83) |
| CD69 | 0.72 (0.23-2.28) | 1.83 (0.58-5.74) | 3.15 (0.79-12.52) | 0.56 (0.11-2.95) | 0.18 (0.02-1.48) | NC | NC | 1.71 (0.26-11.20) | 1.25 (0.21-7.51) |
| TIGIT | 0.46 (0.15-1.41) | 2.95 (0.96-9.08) | 1.03 (0.34-3.15) | 1.26 (0.31-5.05) | 0.30 (0.06-1.52) | 3.90 (0.33-45.66) | NC | 1.23 (0.19-8.00) | NC |
| CD96 | 1.68 (0.56-5.03) | 2.93 (0.90-9.58) | 0.74 (0.24-2.31) | 0.96 (0.22-4.22) | 0.39 (0.08-1.98) | 1.14 (0.10-13.38) | 2.33 (0.14-39.39) | NC | 0.42 (0.05-3.88) |
| **DNAM** | 2.25 (0.72-7.01) | **5.33 (1.34-21.15)*** | 1.79 (0.50-6.42) | 1.16 (0.26-5.13) | 0.86 (0.20-3.64) | NC | NC | 4.61 (0.70-30.44) | 1.37 (0.23-8.25) |
| CD57 | 0.43 (0.12-1.55) | 0.99 (0.31-3.11) | 0.51 (0.16-1.64) | 3.73 (0.91-15.22) | 0.51 (0.10-2.65) | NC | 3.00 (0.18-50.98) | 0.70 (0.07-6.77) | 1.50 (0.25-9.09) |
| **CD85j** | 2.45 (0.85-7.11) | **3.31 (1.07-10.17)*** | 0.83 (0.28-2.49) | 0.37 (0.07-1.91) | 1.27 (0.35-4.59) | NC | NC | 2.77 (0.43-18.01) | 0.83 (0.14-4.95) |
| CRACC | 0.31 (0.08-1.24) | 2.63 (0.73-9.47) | 1.42 (0.39-5.17) | 1.43 (0.32-6.39) | 0.57 (0.11-2.95) | 1.61 (0.13-19.08) | NC | 2.26 (0.34-14.98) | NC |
| 2B4 | 0.93 (0.29-3.01) | 2.02 (0.60-6.73) | 1.60 (0.44-5.78) | 1.29 (0.29-5.71) | 0.95 (0.22-4.05) | 1.47 (0.12-17.36) | 3.00 (0.18-50.98) | 5.08 (0.76-33.71) | 0.55 (0.06-5.07) |
| NTBA | 0.85 (0.25-2.92) | 1.04 (0.31-3.45) | 0.55 (0.16-1.87) | 2.8 (0.66-11.83) | 0.63 (0.12-3.30) | 1.77 (0.15-21.09) | NC | 0.85 (0.09-8.25) | 0.66 (0.07-6.18) |
| CD16 | 0.94 (0.34-2.62) | 0.53 (0.19-1.47) | 0.83 (0.28-2.40) | 1.89 (0.48-7.51) | 1.78 (0.50-6.38) | NC | 1.14 (0.07-19.13) | 0.74 (0.11-4.77) | 0.54 (0.09-3.17) |
| **% NK positive for immunoreceptors ≥p50** | | | | | | | | | |
| NKG2D | 0.38(0.11-1.34) | 3.0 (0.86-10.44) | 1.32 (0.040-4.33) | 2.00 (0.51-7.77) | 0.94 (0.23-3.90) | 0.87 (0.09-8.35) | 3.67(0.22-62.13) | 0.56 (0.06-4.98) | NC |
| NKG2A | 0.67 (0.25-1.79) | 0.76 (0.28-2.03) | 0.99 (0.36-2.73) | 1.74 (0.42-7.10) | 0.95 (0.28-3.21) | 0.78 (0.12-5.03) | 0.52 (0.03-8.71) | 3.51 (0.40-30.95) | 0.27 (0.06-1.25) |
| NKG2C | 0.61 (0.21-1.76) | 1.44 (0.51-4.08) | 0.67 (0.23-1.89) | 0.43 (0.09-2.16) | 0.61 (0.15-2.44) | 0.59 (0.06-5.59) | NC | 0.37 (0.04-3.32) | 0.31 (0.04-2.73) |
| NKP46 | 0.48 (0.18-1.34) | 1.89 (0.70-5.10) | 1.45 (0.52-4.02) | 1.33 (0.37-4.70) | 0.41 (0.10-1.65) | NC | 1.80 (0.11-30.04) | 0.68 (0.12-3.80) | 0.56 (0.10-2.98) |
| **CD69** | **0.32 (0.12-0.87)*** | 1.84 (0.71-4.75) | 2.68 (0.97-7.39) | 1.22 (0.35-4.23) | 0.40 (0.11-1.42) | 0.27 (0.03-2.58) | NC | 0.44 (0.08-2.43) | 0.68 (0.15-3.09) |
| TIGIT | 0.77 (0.28-2.10) | 2.00 (0.72-5.55) | 0.70 (0.25-1.92) | 1.00 (0.27-3.72) | 0.27 (0.06-1.33) | 0.48 (0.05-4.53) | 2.04 (0.12-34.16) | 0.78 (0.14-4.36) | 0.25 (0.03-2.20) |
| CD96 | 1.67 (0.62-4.49) | 1.53 (0.56-4.18) | 0.91 (0.33-2.52) | 1.00 (0.27-3.72) | 0.76 (0.21-2.73) | 1.36 (0.21-8.76) | NC | 0.78 (0.14-4.36) | 1.23 (0.27-5.64) |
| **DNAM** | 0.31 (0.08-1.24) | 2.33 (0.66-8.30) | 1.02 (0.30-3.45) | **4.05 (1.05-15.59)*** | 0.64 (0.13-3.25) | 1.04 (0.11-10.09) | NC | NC | 0.56 (0.06-4.97) |
| CD57 | 0.65 (0.23-1.83) | 1.06 (0.39-2.87) | 0.63 (0.23-1.74) | 1.67 (0.47-5.96) | 0.29 (0.06-1.44) | NC | NC | 0.33 (0.04-2.88) | 2.37 (0.54-10.47) |
| CD85 | 1.74 (0.68-4.49) | 1.79 (0.70-4.58) | 0.92 (0.35-2.40) | 0.71 (0.20-2.50) | 1.07 (0.33-3.44) | 1.64 (0.26-10.46) | NC | 7.45 (0.85-65.41) | 1.89 (0.42-8.58) |
| CRACC | 1.54 (0.51-4.72) | 2.05 (0.63-6.66) | 1.94 (0.55-6.79) | 1.20 (0.28-5.11) | 0.52 (0.10-2.63) | 2.52 (0.38-16.60) | NC | 1.46 (0.25-8.33) | NC |
| **2B4** | **0.20 (0.04-0.99)*** | **5.69 (1.16-27.92)*** | 2.13 (0.53-8.57) | 1.67 (0.38-7.27) | 0.29 (0.03-2.48) | NC | NC | 4.12 (0.80-21.30) | 0.62 (0.07-5.52) |
| NTBA | 0.31 (0.08-1.24) | 1.08 (0.33-3.52) | 1.02 (0.30-3.45) | 1.48 (0.34-6.40) | 0.64 (0.13-3.25) | 1.04 (0.11-10.09) | NC | 0.67 (0.07-6.03) | NC |
| CD16 | 0.86 (0.35-2.08) | 0.83 (0.35-1.98) | 1.39 (0.46-4.20) | 1.60 (0.41-6.23) | 0.42 (0.10-1.76) | NC | NC | NC | 0.72 (0.12-4.25) |

ALL: acute lymphoblastic leukemia; OR: odds ratio; CI: confidence interval; MIF: medium intensity of fluorescence; ≥p50: above 50^th^ percentile; NK: Natural killer; NC: non-calculated; .*:*p*<0.05; **: *p* <0.01; ***: *p* <0.001

(cont´d). Association between NK cells features and clinical variables of pediatric patients with acute lymphoblastic leukemia (n=72)

| **NK cells feature** | **Age >= 10**  *n=*29  OR (IC 95%) | **Sex Male**  *n=*40  OR (IC 95%) | **Infection during the first year of life**  **n=46**  OR (IC 95%) | **Allergy history**  **n=12**  OR (IC 95%) | **Leukocyte count >=50,000**  *n=*14  OR (IC 95%) | **Rearrangement**  *n=*5  OR (IC 95%) | **Immunophenotype T cell**  *n=*2  OR (IC 95%) | **Relapse**  *n=7*  OR (IC 95%) | **Death**  *n=8*  OR (IC 95%) |
| --- | --- | --- | --- | --- | --- | --- | --- | --- | --- |
| **% Subpopulations** ≥p50 | | | | | | | | | |
| NK Dim | 1.43 (0.19-10.75) | 2.51 (0.25-25.40) | NC | NC | 1.41 (0.14-14.68) | NC | NC | NC | NC |
| NK Bright | 1.52 (0.47-4.91) | 3.67 (0.93-14.52) | 0.70 (0.21-2.30) | 2.50 (0.63-9.93) | 0.64 (0.13-3.25) | 3.06 (0.46-20.33) | NC | 0.67 (0.07-6.03) | 0.56 (0.06-4.97) |
| NK Total | 0.68 (0.12-3.97) | 4.43 (0.49-40.00) | NC | NC | 0.81 (0.09-7.59) | 3.10 (0.29-33.27) | NC | NC | NC |
| NKG2A- CD57- | 0.82 (0.29-2.35) | 0.97 (0.35-2.70) | 0.75(0.26-2.21) | 0.98 (0.25-3.92) | 1.07 (0.30-3.85) | 3.13 (0.27-36.52) | NC | 0.99 (0.15-6.37) | 1.54 (0.29-8.36) |
| NKG2A+ CD57- | 1.30 (0.45-3.80) | 0.75 (0.26-2.14) | 0.63 (0.20-1.97) | 0.53 (0.13-2.07) | 1.23 (0.32-4.63) | 1.19 (0.10-13.89) | 0.58 (0.03-9.72) | 0.87 (0.13-5.67) | 0.26 (0.04-1.53) |
| NKG2A+ CD57+ | 1.06 (0.38-2.96) | 1.44 (0.52-3.94) | 0.67 (0.23-1.96) | 1.39 (0.35-5.50) | 0.56 (0.16-2.01) | 1.81 (0.15-21.02) | NC | 1.35 (0.21-8.70) | 0.40 (0.07-2.38) |
| NKG2A- CD57+ | 1.92 (0.68-5.46) | 1.68 (0.59-4.77) | 1.02 (0.34-3.02) | 0.98 (0.25-3.92) | 0.69 (0.18-2.60) | NC | 1.50 (0.09-25.15) | 2.39 (0.37-15.44) | 9.00 (0.98-82.50)* |

ALL: acute lymphoblastic leukemia; OR: odds ratio; CI: confidence interval; MIF: medium intensity of fluorescence; ≥p50: above 50^th^ percentile; NK: Natural killer; NC: non-calculat; *:*p*<0.05; **: *p* <0.01; ***: *p* <0.001
